# Association of reproductive history with breast tissue characteristics and receptor status in the normal breast

**DOI:** 10.1007/s10549-018-4768-0

**Published:** 2018-03-30

**Authors:** Marike Gabrielson, Flaminia Chiesa, Catharina Behmer, Katarina Rönnow, Kamila Czene, Per Hall

**Affiliations:** 1 Department of Medical Epidemiology and Biostatistics, Karolinska Institutet, Nobels väg 12A, 171 77 Stockholm, Sweden; 2 Department of Mammography, Unilabs, Jan Waldenströms gata 22, 205 02 Malmö, Sweden; 3 Department of Mammography, Unilabs, Hospital of Helsingborg, 251 87, Helsingborg, Sweden; 4 Department of Oncology, South General Hospital, 118 83 Stockholm, Sweden

**Keywords:** Breast tissue histology, Reproductive history, Reproductive score, Epithelial breast tissue, Hormone receptor status, Proliferation

## Abstract

**Introduction:**

Reproductive history has been associated with breast cancer risk, but more knowledge of the underlying biological mechanisms is needed. Because of limited data on normal breast tissue from healthy women, we examined associations of reproductive history and established breast cancer risk factors with breast tissue composition and markers of hormone receptors and proliferation in a nested study within the Karolinska Mammography project for risk prediction for breast cancer (Karma).

**Materials and methods:**

Tissues from 153 women were obtained by ultrasound-guided core needle biopsy as part of the Karma project. Immunohistochemical staining was used to assessed histological composition of epithelial, stromal and adipose tissue, epithelial and stromal oestrogen receptor (ER) and progesterone receptor (PR) status, and Ki-67 proliferation status. An individualised reproductive score including parity, number of pregnancies without birth, number of births, age at first birth, and duration of breastfeeding, was calculated based on self-reported reproductive history at the time of the Karma study entry. All analyses were adjusted for age and BMI.

**Results:**

Cumulated reproductive score was associated with increased total epithelial content and greater expression of epithelial ER. Parity was associated with greater epithelial area, increased epithelial–stromal ratio, greater epithelial ER expression and a lower extent of stromal proliferation. Increasing numbers of pregnancies and births were associated with a greater epithelial area in the entire study set, which remained significant among postmenopausal women. Increasing numbers of pregnancies and births were also associated with a greater expression of epithelial ER among postmenopausal women. Longer duration of breastfeeding was associated with greater epithelial area and greater expression of epithelial PR both in the entire study set and among postmenopausal women. Breastfeeding was also positively associated with greater epithelial ER expression among postmenopausal women. Prior use of oral contraceptives was associated with lower epithelial–stromal ratio amongst all participants and among pre- and postmenopausal women separately.

**Conclusion:**

Reproductive risk factors significantly influence the epithelial tissue compartment and expression of hormone receptors in later life. These changes remain after menopause. This study provides deeper insights of the biological mechanisms by which reproductive history influences epithelial area and expression of hormone receptors, and as a consequence the risk of breast cancer.

**Electronic supplementary material:**

The online version of this article (10.1007/s10549-018-4768-0) contains supplementary material, which is available to authorized users.

## Introduction

Reproductive history, like parity, age at first birth and number of births, has consistently been shown to be associated with breast cancer risk [1]. Women who have undergone a full time pregnancy before 20 years of age, for example, have a 50% reduced lifetime risk of developing breast cancer when compared to nulliparous women [2]. Following sex and age, mammographic density is considered one of the strongest risk factors for female breast cancer [3–6]. Mammographic density reflects the composition of supporting connective- (stromal), glandular epithelial- and adipose tissue of the breast. Reproductive history influences the mammographic density [7], thus suggesting a potential mechanism for the effect on risk by breast tissue composition alteration.

During puberty, pregnancy and postmenopausal involution, important shifts occur in normal breast tissue composition in response to changing hormone levels [8–12]. Epithelial proportions increase with hormonal exposure during pregnancy and lactation, whereas menopausal involution of the breast is associated with regression of lobules and relative increase of adipose tissue [8, 13]. Although the underlying biology behind reproductive risk factors and breast cancer risk is not fully understood, proposed mechanisms include increased maturation of the breast parenchyma, greater differentiation of epithelial cells, and change in oestrogen responsiveness of the mammary gland [14]. The frequency of epithelial cells expressing the proliferation marker Ki-67 has also been positively associated with breast cancer risk among premenopausal women [15]. It remains inconclusive however, if parity and reproductive history alter epithelial proliferation [16–18].

A few studies, with conflicting results, have investigated how reproductive behaviour influences oestrogen receptor (ER) and progesterone receptor (PR) expression in normal mammary tissue [19–22]. Changing hormone levels caused by reproductive history may also influence the stromal composition, but previous studies show inconsistent results [8, 23–25]. We have previously found associations between epithelial and stromal hormone receptors and breast tissue characteristics, thus supporting the idea of hormonal regulation of tissue composition [26].

To understand the mechanisms by which reproductive history influences breast cancer risk, better knowledge of how these factors influence the breast tissue composition is needed. In this study, we examined associations of reproductive history and established breast cancer risk factors with breast tissue composition and markers of hormone receptors and proliferation in a nested study within the Karolinska Mammography project for risk prediction for breast cancer (Karma) [27].

## Materials and methods

### Study population and tissue samples

Karma (Karolinska Mammography project for risk prediction for breast cancer) is a population-based prospective cohort study initiated in January 2011 and comprises 70,877 women attending mammography screening or clinical mammography at four hospitals in Sweden [27, 28]. Women participating in the Karma project were recruited for this study. The methods used to collect this material have been described in details elsewhere by Gabrielson et al. [26, 28] and will be given only in brief here. In total, 153 healthy women without prior history of breast cancer, other cancer or breast surgery, were included in the study. Characteristics of all participants are found in Table 1. Information on risk factors and exposures were collected by questionnaire at study enrolment [28]. BMI was calculated based on self-reported height and weight, and was missing for two participants. Areas of dense breast tissue were located by ultrasound and core needle biopsies were extracted from the densest part of the left breast. Tissues were formalin fixed and paraffin embedded. All participants signed an informed consent and the ethical review board at Karolinska Institutet approved the study.

**Table 1:** Characteristics of study participants in a nested biopsy study within the Karolinska mammography project for risk prediction for breast cancer (Karma) (*N *= 153)

Characteristics or histological markers	*N*	Mean (SD), range, or number (%)
Characteristic		
Age at biopsy (y)	153	57.2 (9.0), 41–76
BMI (Kg/m^2^)	151	25.5 (4.4), 18.9–44.8
Age at menarche (y)	145	13.1 (1.4), 9–17
Age at first birth (y)	126	26.3 (5.2), 17–46
Age at menopause (y)	79	50.6 (4.7), 35–59
Parous status	146	
Nulliparous		20 (13.7)
Parous		126 (86.3)
Pregnancies (number)	146	2.4 (1.6), 0–8
Births (number)	146	1.8 (1.1), 0–5
Breastfeeding (months)^a^	125	17.1 (10.1), 0–53
Ever taken oral contraceptives	144	
No		24 (16.7)
Yes		120 (83.3)
Postmenopausal status	150	
Premenopausal		55 (36.7)
Postmenopausal		95 (63.3)
Ever taken hormone replacement therapy	148	
No		106 (71.6)
Yes		42 (28.4)
Benign breast disorder	151	
No		111 (73.5)
Yes		40 (26.5)
Tissue distribution and proteins markers		
Epithelial area (%)	153	3.7 (6.0), 0.0–34.2
Stromal area (%)	153	45.9 (28.9), 1.6–100
Adipose area (%)	153	50.4 (30.9), 0.0–98.4
Epithelial Ki-67 (%)	111	2.4 (2.6), 0.0–17.0
Epithelial ER (%)	110	30.4 (11.2), 4–56.8
Epithelial PR (%)	114	16.1 (12.9), 0–57.9
Stromal Ki-67	112	
Negative		78 (69.6)
Positive		34 (30.4)
Stromal ER	112	
Negative		22 (19.6)
Positive		90 (80.4)
Stromal PR	122	
Negative		20 (16.4)
Positive		102 (83.6)

*BMI* body mass index, *ER* oestrogen receptor, *IHC* immunohistochemical, *PR* progesterone receptor, *SD* standard deviation, *y* years

^a^Minimum total duration of breastfeeding (months)

### Immunohistochemical staining

Whole, 4-µm-thick, paraffin embedded breast core needle biopsy sections were analysed using conventional immunohistochemical (IHC) staining. Staining of the slides has been described elsewhere [26]. Briefly, the sections were incubated with primary monoclonal mouse anti-human antibodies, [anti-oestrogen receptor alpha (clone ID5, 1:60), anti-progesterone receptor (clone PgR 363, 1:50), or anti-Ki-67 (clone MIB-1, 1:75)], all from Dako (Dako Pathology, Stockholm, Sweden), in Antibody Diluent (Dako) for 30 min. A positive reaction was detected using 3,3’-diaminobenzidine (DAB) (Dako) and tissues were counterstained with haematoxylin.

### Image analysis

Preparation of slides and image analysis has been described previously [26]. In brief, for each block a single haematoxylin and eosin section was prepared, on which a pathologist examined the tissue to confirm normal histology. All IHC sections were scanned in an Aperio ScanScope XT slide scanning system (Aperio Technologies, USA) at × 40 magnification and blinded before manually read using the ImageScope viewing software. Digital images of the sections were captured using the ScanScope photo tool. Stromal, epithelial and adipose areas were manually selected using the Annotations tool. The total areas of analysis (comprising stromal, epithelial and adipose tissues) generated through the markup images were used to calculate the proportion of each tissue type. Calculations of epithelial and stromal protein expressions were done without knowledge of the different exposures of the study and have been described previously [26]. In brief, per cent epithelial nuclear expression of ER, PR and Ki-67 was assessed manually for each section separately. Likewise, stromal nuclear expression of ER, PR and Ki-67 was manually categorised as positive (≥ 1% positive cells) or negative for each section. Detailed information on tissue and protein characteristics of the cohort is found in Table 1.

### Statistical analysis

Breast cancer risk factors and lifestyle factors included in the study were age at biopsy, BMI, use of oral menopausal hormone therapy, menopausal status (pre- or postmenopausal), previous benign breast disease history (defined as ever having any biopsy or partial removal procedure, with no cancer detected), age at menarche, parity, number of pregnancies, and number of births. Age at first birth and minimum duration of breastfeeding was assessed among parous women. Biopsy-based outcomes were total percentage epithelial and stromal content in the biopsy, total percentage epithelial immunohistological expressions of ER, PR and Ki-67, and positive or negative stromal expression of ER, PR and Ki-67. Linear regression was used to examine the association between established breast cancer risk factors and reproductive history with epithelial, stromal and adipose tissue measures expressed as percentages of the total area of the section, and percentage expressions of epithelial ER, PR and Ki-67 made from the histological sections. To study the balance in tissue composition between the epithelial and the stromal compartment, the ratios of epithelial-to-stromal proportions were evaluated by linear regression.

The assumption of normal distribution was inspected in all analyses and log transformations were made when necessary. All linear regression analyses of epithelial and stromal tissue compartment distributions and epithelial protein expressions were adjusted for age and BMI unless otherwise indicated.

Logistic regression was used to examine the association between established breast cancer risk factors and reproductive history with stromal protein expression. The analyses of stromal protein expressions were adjusted for age.

We created a reproductive score reflecting the reproductive activity of each study participant. The individual score was calculated by summarising the number of reproductive events (range total score 0–13) and it was defined by including the variables parous (no = 0, yes = 1), number of pregnancies without births (range 0–4), number of births (range 0–5), age at first birth (categories: nulliparous = 0; < 20 = 1; 20–25 = 2; > 25 = 3) and minimum duration of breastfeeding (categories: 0 = 0; < group mean = 1; > group mean = 2). Analyses of proportional growth by quartile groups of epithelial area (%) and epithelial ER (%) with reproductive score were assessed using univariate analysis of variance. Linear associations between percentage epithelial area or epithelial ER and reproductive score (continuous) were analysed by linear regression. Odds ratios (ORs) were evaluated using multinomial logistic regression comparing quartile groups of epithelial area or epithelial ER (cut points based on the overall distribution and the lowest 25% as the reference group in all analyses) with reproductive score for all women and stratified by menopausal status.

In secondary analyses, we stratified by menopausal status at biopsy. In total, 55 women were premenopausal at biopsy collection, and 95 were postmenopausal. Menopausal status was missing for three participants.

Two-tailed *p* values were used for all analyses with a *p* value of less than 0.05 considered to be statistically significant. Statistical analyses were performed using SPSS IBM, version 23.

## Results

### Association of risk factors with epithelial area

Distributions of demographics, menstrual and reproductive history, and selected risk factors for breast cancer are described in Table 1. Histological sections from a total of 153 individuals were examined for association of risk factors with the epithelial tissue compartment (Table 2).

**Table 2:** Linear regression analysis of log-transformed epithelial area expressed as percentage of the total area of the section by risk factors, for all women and stratified by menopausal status, adjusted for age and BMI

Variables	Epithelial area (%)^a^			Epithelial area (%)^a^			Epithelial area (%)^b^		
	All women (*N *= 153)			Premenopausal women (*N *= 55)			Postmenopausal women (*N *= 95)		
	*N*	Estimates β (SE)	*p*	*N*	Estimates β (SE)	*p*	*N*	Estimates β (SE)	*p*
Age at biopsy (y)	153	− 0.022 (0.011)	0.049^b^	55	0.010 (0.042)	0.820^a^	95	− 0.011 (0.018)	0.531^b^
BMI (Kg/m^2^)	151	− 0.053 (0.023)	0.026^c^	54	− 0.056 (0.045)	0.214^c^	94	− 0.057 (0.027)	0.038^c^
Age at menarche (y)	144	0.008 (0.075)	0.911	53	0.112 (0.185)	0.421	91	− 0.076 (0.088)	0.390
Age at first birth (y)	126	0.040 (0.021)	0.059	47	0.036 (0.035)	0.314	79	0.042 (0.027)	0.119
Age at menopause (y)			N.A.			N.A.	78	0.037 (0.026)	0.159
Parous status^d^	145	0.558 (0.302)	0.067	54	0.250 (0.606)	0.681	91	0.705 (0.332)	0.037
Pregnancies (number)	145	0.175 (0.064)	0.007	54	0.247 (0.129)	0.063	91	0.141 (0.070)	0.048
Births (number)	145	0.210 (0.096)	0.030	54	0.119 (0.190)	0.534	91	0.270 (0.105)	0.012
Pregnancies without birth (number)	145	0.212 (0.109)	0.054	54	0.406 (0.197)	0.044	91	0.075 (0.128)	0.556
Breastfeeding (months)^e^	120	0.028 (0.011)	0.016	44	0.034 (0.023)	0.147	76	0.026 (0.013)	0.039
Ever taken oral contraceptives^f^	143	− 0.361 (0.287)	0.211	54	− 0.233 (0.854)	0.786	89	− 0.457 (0.283)	0.110
Postmenopausal status^g^	148	− 0.386 (0.326)	0.239			N.A.			N.A.
Ever taken hormone replacement therapy^f^	147	− 0.302 (0.247)	0.233	54		N.D.	93	− 0.303 (0.238)	0.205
Benign breast disorder^f^	150	0.129 (0.231)	0.578	53	0.042 (0.535)	0.937	94	0.255 (0.247)	0.305

*BMI* body mass index, *N.A.* not applicable, *N.D.* not determined, *SE* standard error, *y* years

^a^Log transformed variable

^b^Unadjusted model

^c^Adjusted for age

^d^Parous versus nulliparous

^e^Minimum total duration of breastfeeding (months) among parous women

^f^Ever versus never

^g^Postmenopausal versus premenopausal

**Fig. 1:**
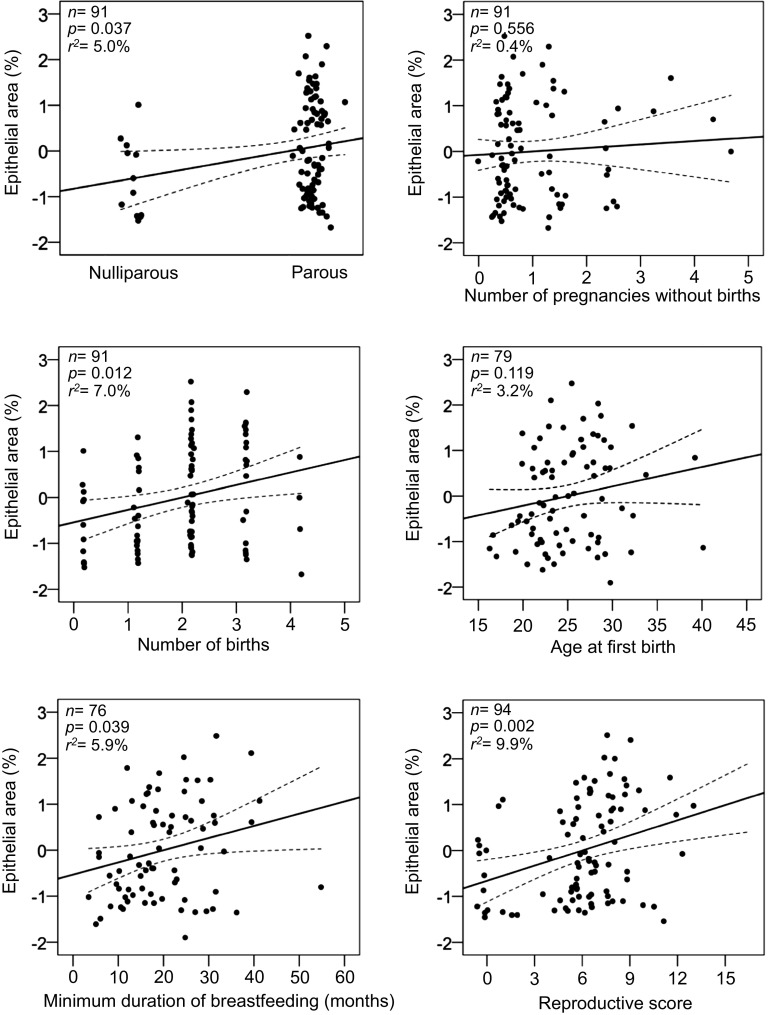
Multivariable regression plots of percentage epithelial tissue (log) by reproductive risk factors and reproductive score in postmenopausal women. The reproductive score was generated by summarising the number of reproductive events defined by parity (no = 0, yes = 1), number of pregnancies without births, number of births, age at first birth (nulliparous = 0, < 20 = 1, 20–25 = 2, > 25 = 3), and minimum duration of breastfeeding (0, < group mean = 1, > group mean = 2). All analyses are adjusted for age and BMI. *X*-axes represent arbitrary numbers of the individual reproductive factors from normalised log-transformed linear regression models

In the total dataset, women at higher age showed smaller epithelial area and the epithelial area seemed to decrease by 2.2% per increasing age at biopsy (*p *= 0.049). Greater BMI was associated with a 5.3% smaller epithelial area per increasing BMI unit (*p *= 0.026), which remained significant among postmenopausal women (*p *= 0.038). Postmenopausal parous women had a 70.5% greater epithelial area compared to nulliparous postmenopausal women (*p *= 0.037) (Table 2). Increasing number of pregnancies, number of births and longer duration of breastfeeding were all associated with a significantly greater epithelial area (17.5% per pregnancy; 21.0% per birth; 2.8% per month; respectively), and remained significantly associated among postmenopausal women (14.1% per pregnancy; 27% per birth; 2.6% per month; respectively) (Table 2, Fig. 1).

To better capture the combined effects of reproductive factors on the epithelial tissue compartment, we created a reproductive score reflecting the reproductive activity of each study participant. Total per cent epithelial area increased by 43.4% (*p *< 0.001) per quartile increase in reproductive score when adjusted for age and BMI in the total data set, which remained similar among postmenopausal women (39.9%; *p *= 0.001) (Supplemental Table S1).

**Table 3:** Multinomial logistic regression of quartiles per cent epithelial area expressed as percentage of the total area of the section and reproductive score for all women and stratified by menopausal status

	Epithelial area (%), quartiles^a^							Linear estimate β (SE)^b^	*p* for trend^b^
	1st	2nd		3rd		4th			
	Reference	OR	95% CI	OR	95% CI	OR	95% CI		
Reproductive score, all women (*N *= 150)^c^	1.0	1.082	0.920–1.273	1.113	0.956–1.296	1.352	1.126–1.622	0.111 (0.032)	0.001
Reproductive score, premenopausal women (*N *= 54)^c^	1.0	0.966	0.732–1.349	1.224	0.890–1.684	1.206	0.887**–** 1.641	0.095 (0.073)	0.198
Reproductive score, postmenopausal women (*N *= 94)^c^	1.0	1.074	0.889–1.297	1.176	0.960–1.441	1.281	1.044–1.571	0.110 (0.035)	0.002

Adjusted for age and BMI

*BMI* body mass index, *OR* odds ratio, *CI* confidence interval

^a^1st, 2nd, 3rd and 4th quartile respectively: ≤ 0.0, 0.1–1.0, 1.1–4.6, > 4.7%

^b^Determined using log-transformed epithelial area as continuous variable

^c^Variables included in the reproductive score: parous (no = 0, yes = 1), number of pregnancies without birth (0–4), number of births (0–5), age at first birth (nulliparous = 0, < 20 = 1, 20–25 = 2, > 25 = 3), and minimum duration of breastfeeding (0, < group mean = 1, > group mean = 2)

Among all women, a higher reproductive score was positively associated with a 11.1% greater epithelial content per unit increase (*p* for trend = 0.001) (Table 3), with the OR in the highest quartile compared with the lowest quartile being 1.35 (95% CI 1.13–1.62) when adjusting for age and BMI. The findings remained significant for postmenopausal women (11.0% increase per unit reproductive score) (*p* for trend = 0.002) (Table 3, Fig. 1), with the OR in the highest quartile compared with the lowest quartile being 1.28 (95% CI 1.04–1.57).

### Association of risk factors with epithelial protein expression

**Fig. 2:**
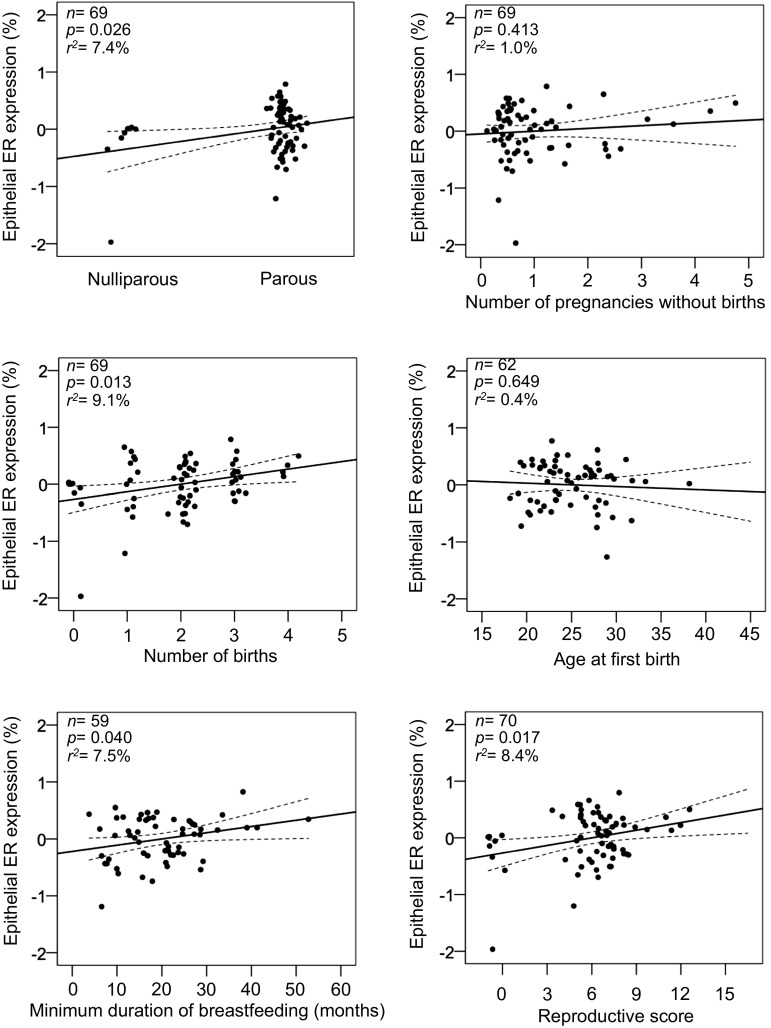
Multivariable regression plots of percentage epithelial oestrogen receptor (ER) expression (log) by reproductive risk factors and reproductive score in postmenopausal women. The reproductive score was generated by summarising the number of reproductive events defined by parity (no = 0, yes = 1), number of pregnancies without births, number of births, age at first birth (nulliparous = 0, < 20 = 1, 20–25 = 2, > 25 = 3), and minimum duration of breastfeeding (0, < group mean = 1, > group mean = 2). All analyses are adjusted for age and BMI. *X*-axes represent arbitrary numbers of the individual reproductive factors from normalised log-transformed linear regression models

In the total data set, higher age was significantly associated with a 1.0% higher expression of ER per year increase at biopsy (*p *= 0.033) (Table 4) and a 2.2% lower expression of PR per year (*p *= 0.049) (Supplemental Table S2). Furthermore, higher BMI was associated with a significantly higher expression of PR (6.4%; *p *= 0.041). Increasing number of births was significantly associated with greater expression of ER in the total data set (10.0% per birth; *p *= 0.017), which remained significant among postmenopausal women (13.2% per birth; *p *= 0.013) (Table 4, Fig. 2). Parous postmenopausal women also had 40.8% higher ER expression (*p *= 0.026), and greater ER by increasing number of pregnancies (7.4% per pregnancy; *p *= 0.035). Minimum duration of breastfeeding was positively associated with a 3.2% higher expression of PR in all women per additional month of breastfeeding (*p *= 0.003), with similar results among postmenopausal women (4.4%; *p *= 0.004). Among postmenopausal women, breastfeeding was also associated with greater ER (1.1%; *p *= 0.040).

**Table 4:** Linear regression analysis of epithelial ER and risk factors, for all women and stratified by menopausal status, adjusted for age and BMI

Variables	Epithelial ER (%)^a^			Epithelial ER (%)^a^			Epithelial ER (%)^a^		
	All women (*N *= 153)			Premenopausal women (*N *= 55)			Postmenopausal women (*N *= 95)		
	*N*	Estimates β (SE)	*p*	*N*	Estimates β (SE)	*p*	*N*	Estimates β (SE)	*p*
Age at biopsy (y)	110	0.010 (0.005)	0.033^b^	37	0.006 (0.016)	0.734^b^	71	0.012 (0.009)	0.180^b^
BMI (Kg/m^2^)	108	0.025 (0.014)	0.082^c^	36	0.015 (0.020)	0.445^c^	70	0.041 (0.022)	0.064^c^
Age at menarche (y)	103	− 0.042 (0.030)	0.167	35	− 0.030 (0.053)	0.581	68	− 0.851 (0.989)	0.392
Age at first birth (y)	94	− 0.018 (0.009)	0.051	32	− 0.030 (0.013)	0.025	62	− 0.006 (0.013)	0.649
Age at menopause (y)			N.A.			N.A.	61	− 0.015 (0.012)	0.221
Parous status^d^	105	0.250 (0.142)	0.081	36	0.079 (0.247)	0.750	69	0.408 (0.179)	0.026
Pregnancies (number)	105	0.053 (0.027)	0.051	36	0.018 (0.045)	0.698	69	0.074 (0.034)	0.035
Births (number)	105	0.100 (0.041)	0.017	36	0.044 (0.070)	0.535	69	0.132 (0.052)	0.013
Pregnancies without birth (number)	105	0.030 (0.045)	0.503	36	− 0.001 (0.072)	0.985	69	0.049 (0.059)	0.413
Breastfeeding (months)^e^	88	0.007 (0.004)	0.120	29	− 0.002 (0.007)	0.785	59	0.011 (0.005)	0.040
Ever taken oral contraceptives^f^	103	0.104 (0.121)	0.394	36	0.125 (0.337)	0.714	67	0.093 (0.133)	0.489
Postmenopausal status^g^	106	0.024 (0.146)	0.868			N.A.			N.A.
Ever taken hormone replacement therapy^f^	105	− 0.009 (0.109)	0.933	36		N.D.	69	0.060 (0.117)	0.609
Benign breast disorder^f^	107	0.022 (0.098)	0.824	35	0.094 (0.204)	0.650	70	− 0.004 (0.119)	0.971

*BMI* body mass index, *ER* oestrogen receptor, *N.A.* not applicable, *N.D.* not determined, *SE* standard error, *y* years

^a^Log transformed variable

^b^Unadjusted model

^c^Adjusted for age

^d^Parous versus nulliparous

^e^Minimum total duration of breastfeeding (months) among parous women

^f^Ever versus never

^g^Postmenopausal versus premenopausal

In the total data set, a higher reproductive score was positively associated with a 3.0% greater epithelial ER expression per unit increase (*p* for trend = 0.043), with the OR in the highest quartile of reproductive score (median ER 43.0%) compared with the lowest quartile (median ER 16.1%) being 1.29 (95% CI 1.04–1.61), when adjusting for age and BMI. When stratifying be menopausal status, a higher reproductive score was positively associated with 4.5% greater epithelial ER expression per unit increase (*p* for trend = 0.017) (OR 1.239; 95% CI 0.953–1.612 for the highest quartile) when adjusting for age and BMI (Table 5, Fig. 2) among postmenopausal women. We did not see any significant associations between reproductive score and epithelial ER among premenopausal women.

**Table 5:** Multinomial logistic regression of quartiles per cent epithelial ER and reproductive score for all women and stratified by menopausal status

	Epithelial ER (%), quartiles^a^							Linear estimate β (SE)^b^	*p* for trend^b^
	1st	2nd		3rd		4th			
	(Reference)	OR	95% CI	OR	95% CI	OR	95% CI		
Reproductive score, all women (*N *= 107)^c^	1.0	1.263	1.017–1.570	1.072	0.892–1.289	1.290	1.036–1.605	0.030 (0.015)	0.043
Reproductive score, premenopausal women (*N *= 36)^c^	1.0	1.079	0.769–1.515	1.596	0.930–2.740	1.032	0.753–1.413	0.006 (0.027)	0.818
Reproductive score, postmenopausal women (*N *= 70)^c^	1.0	1.158	0.908–1.476	1.211	0.947–1.550	1.239	0.953–1.612	0.045 (0.018)	0.017

Adjusted for age and BMI

*BMI* body mass index, *ER* oestrogen receptor, *OR* odds ratio, *CI* confidence interval

^a^1st, 2nd, 3rd and 4th quartile, respectively: ≤ 23.6, 23.7–30.6, 30.7–38.6, > 38.7%

^b^Determined using log-transformed epithelial ER as continuous variables

^c^Variables included in the reproductive score: parous (no = 0, yes = 1), number of pregnancies without birth (0–4), number of births (0–5), age at first birth (nulliparous = 0, < 20 = 1, 20–25 = 2, > 25 = 3), and minimum duration of breastfeeding (0, < group mean = 1, > group mean = 2)

Amongst premenopausal women, we found no significant associations between any of the risk factors and epithelial hormone receptor expressions (Table 4 and Supplemental Table S2). Likewise, we found no significant associations between any of the risk factors and epithelial Ki-67 (Supplemental Table S3).

### Association of risk factors with stromal compartment and stromal protein expression

The only risk factor significantly associated with stromal area in our study was BMI, which was associated with a smaller stromal area among all women (*p *< 0.001) (Supplemental Table S4). This finding remained significant after stratifying into premenopausal and postmenopausal women (*p *= 0.002) and (*p *= 0.035), respectively. We also investigated the epithelial–stromal ratio in our cohort. In the total dataset, increasing age was associated with a reduction of the epithelial–stromal ratio (*p *= 0.008) (Supplemental Table S5). Higher age at first birth was associated with a greater epithelial–stromal ratio in the total cohort (*p *= 0.004) and among premenopausal women (*p *= 0.012). Among postmenopausal women, parous women had a greater epithelial–stromal ratio (*p *= 0.036); furthermore, greater epithelial–stromal ratio was associated with higher number of births (*p *= 0.021). Prior use of oral contraceptives was associated with lower epithelial–stromal ratio amongst all participants and after stratification by menopausal status (total, *p *= 0.020; premenopausal, *p *= 0.034; and postmenopausal, *p *= 0.028).

In the total dataset, nulliparous women more frequently exhibited positive stromal expression of Ki-67 (*p *= 0.040). Likewise, higher BMI was associated with expression of stromal Ki-67 among postmenopausal women (*p *= 0.030). Lack of stromal PR was associated with higher age at first birth among all women (*p *= 0.012), and in postmenopausal women (*p* = 0.042).

## Discussion

We found that reproductive history significantly influences the epithelial tissue content and expression of hormone receptors in later life and that these changes remain after menopause. Specifically, our results show that parity, number of pregnancies, number of births and duration of breastfeeding all were individually associated with a greater proportion of epithelial tissue after adjusting for age and BMI. Consistently, we found a strong positive relationship between epithelial content and the reproductive score we created reflecting the combined effects of reproductive history.

The human breast is defined by the degree of complexity of the secretory lobules, generally categorised as type 1–4, in the order of increasing complexity [10, 12, 29], although the cut points defining lobule types remain under discussion. The non-cancerous breast of parous women contains more type 3 lobules with a concomitant reduction in type 1 lobules [10]. Parous women also have more terminal duct lobular units (TDLU), and an increasing number of live births is associated with increasing TDLU counts [30]. Intuitively, the dramatically increased exposure to female sex hormones during each pregnancy could increase epithelial proliferation and thus the total amount of epithelial glandular tissue. To further support our findings, greater epithelial proportions are associated with parity and number of births in age-adjusted models [8]. Other studies, however, have not found any associations between reproductive history and glandular area from forensic autopsies [23] or epithelial content in non-neoplastic adjacent breast tissues from invasive breast tumours [25]. We did not investigate lobule types in this study, although we would expect that the epithelial proportion would increase with more complex branching lobule types, which by definition contain greater amounts of epithelia.

A few studies, with conflicting results, have evaluated associations between parity and/or reproductive risk factors and expression of ER in human breast tissues [19–22]. The heterogeneity between results in the previous studies and the present one may reflect different statistical approaches and number of observations, menopausal status of subjects, and the type of tissue samples used. Expression of ER in TDLUs in near proximity to breast tumours has been positively associated with ER expression in the tumour, as compared with TDLUs further away [31]. Field effects surrounding the breast cancer may thus influence ER expression, and should be taken into consideration when analysing benign epithelium adjacent to the tumour. We only included non-diseased women without prior history of breast cancer or other cancer in our study. Further studies are needed to determine the relationship between parity and reproductive history with epithelial expression of ER in the normal human epithelium.

While expression of ER in benign epithelium was initially associated with breast cancer risk [20], recent studies have failed to confirm this [15, 31–33]. Circulating oestradiol levels and ER expression in normal epithelium is inversely associated [31, 34], and parity and number of births are associated with lower concentrations of circulating oestrogens [35, 36]. These findings suggest that the positive association between reproductive history and epithelial ER expression found in our study more likely reflects lower levels of circulating oestrogens rather than a direct causal relationship between parity and epithelial ER. Interestingly, although epithelial ER expression was associated with reproductive history, we found no association between reproductive history and epithelial proliferation after adjusting for age and BMI. In fact, epithelial proliferation is inversely associated with ER [34], and the frequency of Ki-67-expressing cells has been positively associated with increased risk of breast cancer, where high Ki-67+/low ER+ cell frequency was significantly associated with a 4.5-fold higher risk of breast cancer compared to low Ki-67+/high ER+ cell frequencies [15]. Collectively, this suggests that epithelial expression of ER without an increased mitotic activity does not mediate increased risk; rather it is a cumulative effect reflecting lower levels of circulating oestrogens as a consequence of parity and reproductive history. Likewise, and in agreement with previous findings, the positive association between reproductive factors protective of breast cancer and epithelial area without a corresponding increase of epithelial proliferation suggests that a greater epithelial area per se does not mediate an increased risk. Parity-associated protection against breast cancer may thus be mediated through non-breast epithelial tissue specific mechanisms, such as reduced endogenous hormone levels through exhausted ovaries.

Duration of breastfeeding was associated with increased epithelial area later in life in our overall study population and among postmenopausal women. These findings are in agreement with that of Figueroa et al. [30] who found a positive association between ever having breastfed and the number of TDLUs in postmenopausal women, and others who found positive associations between fibroglandular tissue and duration of lactation [24, 37]. On the other hand, our results conflict with those studies that found no association [38, 39]. We did not observe any significant changes in stromal compartment associated with duration of breastfeeding, however; amount of adipose tissue was inversely associated with breastfeeding (Supplemental information). Duration of breastfeeding was also positively associated with epithelial expression of PR in our overall study population and among postmenopausal women. Breastfeeding is protective for the luminal A, B and basal-like subtypes of breast cancer, with the largest protective effect of breastfeeding on the risk of the latter [40]. Basal-like cancers are thought to originate from undifferentiated luminal progenitor cells [41]. Fully differentiated type 4 lobules do not form until the end of pregnancy and during lactation when the breast attains its maximum development and increase in lobule numbers and size [42]. Progesterone is necessary for the lobuloalveolar mammary gland development [43, 44], and the luminal population of epithelial cells express more PR compared to the stem cell-enriched fraction [45, 46]. After menopause, the type 4 lobules regress to predominantly type 1-lobules, although more differentiated [47]. Data also suggest that breastfeeding is associated with less involution in postmenopausal women [30]. Although we did not determine lobule types and differentiation in this study, we hypothesise that epithelial expression of PR would increase with more complex and differentiated branching lobule types.

Mammographic density is inversely associated with reproductive history [48], suggesting that reproductive factors also influence the stromal compartment. Although we did not detect any significant associations between stromal content and reproductive history in this study, parous women tended to have lesser stroma compared to nulliparous women. Similarly, parity and a higher reproductive score were non-significantly associated with lower mammographic density, with the greatest effect observed among postmenopausal women (data not shown). Parity was, however, significantly associated with lower expression of Ki-67 in stromal cells, and increased epithelial–stromal ratio in postmenopausal women only, further substantiating the hypothesis of reproductive history-associated regulation of the stromal content. Notably, although we observed stromal expression of ER, we did not find any associations between stromal ER and reproductive history after adjusting for age.

Collectively, our results suggest that while both the epithelial and stromal compartment express ER they are differentially responsive to oestrogen. More studies are needed in this field to clarify through which mechanisms parity regulate the stromal compartment. To the best of our knowledge, this is the first study investigating how parity influences stromal proliferation.

Strengths of our study include analysis of normal breast tissues from women without any history of breast cancer, quantitative measures of breast tissue composition, ER, PR and Ki-67 expression, use of standardised and optimised sample collection procedures, and a rich collection of epidemiologic data through the Karma cohort. Although our study is relatively small, it is among the largest exploring relationships between breast cancer risk factors, tissue characteristics and marker expression in normal breast tissue. However, the restricted number of samples in stratified analyses, particularly among premenopausal women, may bias our results. Also, all epidemiological data were self-reported at the Karma study entry, which may lead to misreporting and missing information. All biopsies included in this study were ultrasound-guided and collected in the densest part of the breast, which may limit the generalizability of findings. Nonetheless, we have previously shown that these tissues are representative of breast tissue composition in the normal breast [26].

In conclusion, this study provides deeper insights of the biological mechanisms by which reproductive history influences the risk of breast cancer. Specifically, our results show that several reproductive risk factors significantly influence breast tissue composition through the epithelial tissue compartment and expression of hormone receptors in later life, and that these changes remain after menopause. Association of risk reducing reproductive risk factors with greater epithelial area and increased expression of ER, but without increased mitotic activity, suggest that these morphologic changes alone do not contribute to an increased risk of breast cancer.

## Electronic supplementary material

Below is the link to the electronic supplementary material.

Supplementary material 1 (DOCX 57 kb)
