# Supplementary material for: Association of reproductive history with breast tissue characteristics and receptor status in the normal breast
Source: Breast Cancer Res Treat. 2018 Mar 30;170(3):487–97. doi: 10.1007/s10549-018-4768-0 (PMC6022521; doi:10.1007/s10549-018-4768-0)
Supplement: Supplementary file 1 — Supplementary material 1 (DOCX 57 kb) [file 10549_2018_4768_MOESM1_ESM.docx]

**Supplemental Tables**

**Supplemental Table S1.** Analysis of variance of associations^a^ between epithelial area (expressed as percentage of the total area of the section) and epithelial ER receptor and reproductive score, for all women and stratified by menopausal status.

|  | Reproductive score, quartiles^b^ | | | |  |  |
| --- | --- | --- | --- | --- | --- | --- |
|  | 1^st^ | 2^nd^ | 3^rd^ | 4^th^ | % increase per quartile^c^ | *p*-value trend^c^ |
| Epithelial area, all women  (Geometric mean^a^, *N*=150) | 1.23 | 1.17 | 2.72 | 2.79 | 43.4 | **<0.001** |
| Epithelial area, premenopausal women  (Geometric mean^a^, *N*=54) | 2.68 | 1.53 | 5.55 | 3.62 | 61.7 | 0.166 |
| Epithelial area, postmenopausal women  (Geometric mean^a^, *N*=94) | 0.93 | 1.42 | 2.23 | 2.45 | 39.9 | **0.001** |
|  |  |  |  |  |  |  |
| Epithelial ER, all women  (Geometric mean^a^, *N*=107) | 24.26 | 28.76 | 28.25 | 29.61 | 7.2 | 0.207 |
| Epithelial ER, premenopausal women  (Geometric mean^a^, *N*=36) | 25.53 | 26.58 | 23.41 | 24.93 | -0.4 | 0.737 |
| Epithelial ER, postmenopausal women  (Geometric mean^a^, *N*=70) | 23.36 | 29.93 | 30.23 | 32.39 | 12.1 | 0.098 |
|  |  |  |  |  |  |  |

^a^ adjusted for age and BMI at blood draw and time of day of blood draw.

^b^ Variables included in the reproductive score: parous (no=0, yes=1), number of pregnancies without birth (0-4), number of births (0-5), age at first birth (nulliparous=0, <20=1, 20-25=2, >25=3), and minimum duration of breast-feeding (0, <group mean=1, >group mean=2).

^c^ Average percentage change in epithelial area or epithelial ER level per quartile increase in reproductive score. *P*-value based on ANOVA.

**Supplemental Table S2.** Linear regression analysis of log-transformed epithelial PR receptor and risk factors, for all women and stratified by menopausal status, adjusted for age and BMI.

|  | Epithelial PR (%)*  All women (*N*=153) | | |  | Epithelial PR (%)*  Premenopausal women (*N*=55) | | |  | Epithelial PR (%)*  Postmenopausal women (*N*=95) | | |
| --- | --- | --- | --- | --- | --- | --- | --- | --- | --- | --- | --- |
| Variables | *n* | Estimates β (SE) | *P* |  | *n* | Estimates β (SE) | *P* |  | *n* | Estimates β (SE) | *P* |
| Age at biopsy (y) | 114 | **-0.022 (0.011)** | **0.049**^a^ |  | 40 | -0.042 (0.026) | 0.116^a^ |  | 72 | 0.012 (0.022) | 0.579^a^ |
| BMI (Kg/m^2^) | 112 | **0.064 (0.031)** | **0.041**^b^ |  | 39 | 0.037 (0.029) | 0.208^b^ |  | 71 | 0.082 (0.054) | 0.128^b^ |
| Age at menarche (y) | 107 | -0.021 (0.071) | 0.773 |  | 38 | -0.001 (0.089) | 0.995 |  | 69 | -0.048 (0.099) | 0.627 |
| Age at first birth (y) | 97 | 0.014 (0.021) | 0.522 |  | 35 | -0.019 (0.021) | 0.369 |  | 62 | 0.065 (0.036) | 0.079 |
| Age at menopause (y) |  |  | *N.A.* |  |  |  | *N.A.* |  | 61 | -0.053 (0.029) | 0.075 |
| Parous status^¶^ | 109 | -0.236 (0.312) | 0.452 |  | 39 | -0.392 (0.409) | 0.344 |  | 70 | -0.079 (0.430) | 0.854 |
| Pregnancies (number) | 109 | 0.047 (0.058) | 0.418 |  | 39 | -0.054 (0.065) | 0.412 |  | 70 | 0.135 (0.085) | 0.116 |
| Births (number) | 109 | 0.052 (0.082) | 0.528 |  | 39 | -0.033 (0.087) | 0.705 |  | 70 | 0.147 (0.129) | 0.262 |
| Breast-feeding (months)^◊^ | 91 | **0.032 (0.010)** | **0.003** |  | 32 | 0.014 (0.013) | 0.302 |  | 59 | **0.044 (0.015)** | **0.004** |
| Ever taken oral contraceptives^‡^ | 107 | 0.303 (0.271) | 0.266 |  | 39 | 0.423 (0.561) | 0.456 |  | 68 | 0.275 (0.326) | 0.403 |
| Postmenopausal status^§^ | 110 | -0.424 (0.325) | 0.195 |  |  |  | *N.A.* |  |  |  | *N.A.* |
| Ever taken hormone replacement therapy^‡^ | 108 | -0.451 (0.247) | 0.071 |  | 38 |  | *N.D.* |  | 70 | -0.487 (0.289) | 0.097 |
| Benign breast disorder^‡^ | 111 | -0.005 (0.229) | 0.984 |  | 38 | 0.208 (0.339) | 0.544 |  | 71 | 0.106 (0.299) | 0.723 |

Abbreviations: BMI, body mass index; N.A., not applicable; N.D., not determined; PR, progesterone receptor; SE, standard error; y, years.

* Log transformed variable

^a^ Unadjusted model

^b^ Adjusted for age

^¶^ Parous versus nulliparous

^◊^ Minimum total duration of breast-feeding (months) among parous women

^‡^ Ever versus never

^§^ Postmenopausal versus premenopausal

**Supplemental Table S3.** Linear regression analysis of log-transformed epithelial Ki-67 and risk factors, for all women and stratified by menopausal status, adjusted for age and BMI.

|  | Epithelial Ki-67 (%)*  All women (*N*=153) | | |  | Epithelial Ki-67 (%)*  Premenopausal women (*N*=55) | | |  | Epithelial Ki-67 (%)*  Postmenopausal women (*N*=95) | | |
| --- | --- | --- | --- | --- | --- | --- | --- | --- | --- | --- | --- |
| Variables | *n* | Estimates β (SE) | *P* |  | *n* | Estimates β (SE) | *P* |  | *n* | Estimates β (SE) | *P* |
| Age at biopsy (y) | 111 | -0.014 (0.008) | 0.101^a^ |  | 39 | -0.006 (0.033) | 0.855^a^ |  | 70 | -0.005 (0.014) | 0.712^a^ |
| BMI (Kg/m^2^) | 109 | 0.007 (0.024) | 0.781^b^ |  | 38 | -0.019 (0.037) | 0.613^b^ |  | 69 | 0.027 (0.035) | 0.431^b^ |
| Age at menarche (y) | 104 | -0.042 (0.056) | 0.462 |  | 37 | -0.045 (0.124) | 0.715 |  | 67 | -0.058 (0.061) | 0.346 |
| Age at first birth (y) | 96 | 0.012 (0.016) | 0.453 |  | 35 | -0.001 (0.024) | 0.955 |  | 61 | 0.034 (0.023) | 0.143 |
| Age at menopause (y) |  |  | *N.A.* |  |  |  | *N.A.* |  | 60 | 0.032 (0.020) | 0.112 |
| Parous status^¶^ | 106 | -0.005 (0.266) | 0.986 |  | 38 | -0.781 (0.603) | 0.204 |  | 68 | 0.331 (0.289) | 0.255 |
| Pregnancies (number) | 106 | -0.009 (0.047) | 0.841 |  | 38 | -0.026 (0.085) | 0.765 |  | 68 | 0.011 (0.056) | 0.840 |
| Births (number) | 106 | -0.005 (0.066) | 0.946 |  | 38 | -0.078 (0.111) | 0.488 |  | 68 | 0.111 (0.085) | 0.195 |
| Breast-feeding (months)^◊^ | 90 | 0.014 (0.008) | 0.086 |  | 32 | 0.024 (0.015) | 0.120 |  | 58 | 0.010 (0.010) | 0.337 |
| Ever taken oral contraceptives^‡^ | 104 | -0.205 (0.215) | 0.341 |  | 38 | 0.504 (0.705) | 0.479 |  | 66 | -0.338 (0.205) | 0.104 |
| Postmenopausal status^§^ | 107 | -0.243 (0.254) | 0.341 |  |  |  | *N.A.* |  |  |  | *N.A.* |
| Ever taken hormone replacement therapy^‡^ | 105 | -0.157 (0.186) | 0.401 |  | 37 |  | *N.D.* |  | 68 | -0.272 (0.177) | 0.129 |
| Benign breast disorder^‡^ | 108 | -0.024 (0.177) | 0.891 |  | 37 | 0.651 (0.449) | 0.156 |  | 69 | -0.179 (0.184) | 0.336 |

Abbreviations: BMI, body mass index; N.A., not applicable; N.D., not determined; SE, standard error; y, years.

* Log transformed variable

^a^ Unadjusted model

^b^ Adjusted for age

^¶^ Parous versus nulliparous

^◊^ Minimum total duration of breast-feeding (months) among parous women

^‡^ Ever versus never

^§^ Postmenopausal versus premenopausal

**Supplemental Table S4.** Linear regression analysis of stromal area expressed as percentage of the total area of the section and risk factors, for all women and stratified by menopausal status, adjusted for age and BMI.

|  |  | Stromal area (%)  All women (*N*=153) | | |  | Stromal area (%)  Premenopausal women (*N*=55) | | |  | Stromal area (%)  Postmenopausal women (*N*=95) | | |
| --- | --- | --- | --- | --- | --- | --- | --- | --- | --- | --- | --- | --- |
| Variables |  | *N* | Estimates β (SE) | *P* |  | *N* | Estimates β (SE) | *P* |  | *N* | Estimates β (SE) | *P* |
| Age at biopsy (y) |  | 153 | 0.265 (0.260) | 0.311^a^ |  | 55 | 1.006 (0.782) | 0.204^a^ |  | 95 | 0.054 (0.492) | 0.912^a^ |
| BMI (Kg/m^2^) |  | 151 | **-1.862 (0.525)** | **0.001^b^** |  | 54 | **-2.386 (0.740)** | **0.002**^b^ |  | 94 | **-1.564 (0.732)** | **0.035**^b^ |
| Age at menarche (y) |  | 144 | -0.002 (1.677) | 0.999 |  | 53 | 3.408 (2.269) | 0.139 |  | 91 | -2.429 (2.356) | 0.305 |
| Age at first birth (y) |  | 126 | 0.304 (0.463) | 0.513 |  | 47 | -0.296 (0.568) | 0.604 |  | 79 | 0.962 (0.718) | 0.185 |
| Age at menopause (y) |  |  |  | *N.A.* |  |  |  | *N.A.* |  | 78 | -0.475 (0.714) | 0.508 |
| Parous status^¶^ |  | 145 | -5.538 (6.845) | 0.420 |  | 54 | -10.968 (9.964) | 0.276 |  | 91 | -3.001 (9.322) | 0.748 |
| Pregnancies (number) |  | 145 | 0.498 (1.473) | 0.736 |  | 54 | 1.022 (2.224) | 0.648 |  | 91 | 0.054 (1.971) | 0.978 |
| Births (number) |  | 145 | 0.005 (2.190) | 0.998 |  | 54 | -0.810 (3.171) | 0.799 |  | 91 | 0.322 (2.993) | 0.915 |
| Breast-feeding (months)^◊^ |  | 120 | 0.429 (0.259) | 0.101 |  | 44 | 0.344 (0.402) | 0.397 |  | 76 | 0.495 (0.343) | 0.154 |
| Ever taken oral contraceptives^‡^ |  | 143 | 4.516 (6.465) | 0.486 |  | 54 | 16.413 (14.023) | 0.247 |  | 89 | 0.311 (7.845) | 0.968 |
| Postmenopausal status^§^ |  | 148 | -0.612 (7.349) | 0.934 |  |  |  | *N.A.* |  |  |  | *N.A.* |
| Ever taken hormone replacement therapy^‡^ |  | 147 | -4.346 (5.573) | 0.437 |  | 54 |  | *N.D.* |  | 93 | -5.118 (6.552) | 0.437 |
| Benign breast disorder^‡^ |  | 150 | -1.789 (5.198) | 0.731 |  | 53 | 1.426 (8.858) | 0.873 |  | 94 | -3.313 (6.735) | 0.624 |

Abbreviations: BMI, body mass index; N.A., not applicable; N.D., not determined; SE, standard error; y, years.

^a^ Unadjusted model

^b^ Adjusted for age

^¶^ Parous versus nulliparous

^◊^ Minimum total duration of breast-feeding (months) among parous women

^‡^ Ever versus never

^§^ Postmenopausal versus premenopausal

**Supplemental Table S5.** Linear regression analysis of log-transformed ratio epithelial to stromal area and risk factors, for all women and stratified by menopausal status, adjusted for age and BMI.

|  |  | Ratio epithelial to stromal area (%)*  All women (*N*=153) | | |  | Ratio epithelial to stromal area (%)*  Premenopausal women (*N*=55) | | |  | Ratio epithelial to stromal area (%)*  Postmenopausal women (*N*=95) | | |
| --- | --- | --- | --- | --- | --- | --- | --- | --- | --- | --- | --- | --- |
| Variables |  | *N* | Estimates β (SE) | *P* |  | *N* | Estimates β (SE) | *P* |  | *N* | Estimates β (SE) | *P* |
| Age at biopsy (y) |  | 153 | **-0.006 (0.002)** | **0.008**^a^ |  | 55 | 0.003 (0.010) | 0.792^a^ |  | 95 | -0.002 (0.002) | 0.278^a^ |
| BMI (Kg/m^2^) |  | 151 | 0.001 (0.005) | 0.901^b^ |  | 54 | 0.006 (0.011) | 0.621^b^ |  | 94 | -0.004 (0.003) | 0.268^b^ |
| Age at menarche (y) |  | 144 | -0.006 (0.015) | 0.685 |  | 53 | -0.018 (0.035) | 0.600 |  | 91 | 0.001 (0.011) | 0.962 |
| Age at first birth (y) |  | 126 | **0.013 (0.004)** | **0.004** |  | 47 | **0.023 (0.009)** | **0.012** |  | 79 | 0.001 (0.003) | 0.762 |
| Age at menopause (y) |  |  |  | *N.A.* |  |  |  | *N.A.* |  | 78 | 0.002 (0.003) | 0.472 |
| Parous status^¶^ |  | 145 | 0.081 (0.061) | 0.188 |  | 54 | 0.051 (0.152) | 0.741 |  | 91 | **0.086 (0.040)** | **0.036** |
| Pregnancies (number) |  | 145 | 0.025 (0.013) | 0.056 |  | 54 | 0.047 (0.033) | 0.164 |  | 91 | 0.017 (0.009) | 0.053 |
| Births (number) |  | 145 | 0.013 (0.020) | 0.499 |  | 54 | -0.010 (0.048) | 0.841 |  | 91 | **0.030 (0.013)** | **0.021** |
| Breast-feeding (months)^◊^ |  | 120 | 0.001 (0.002) | 0.720 |  | 44 | 0.000 (0.006) | 0.953 |  | 76 | 0.002 (0.002) | 0.185 |
| Ever taken oral contraceptives^‡^ |  | 143 | **-0.135 (0.057)** | **0.020** |  | 54 | **-0.447 (0.205)** | **0.034** |  | 89 | **-0.076 (0.034)** | **0.028** |
| Postmenopausal status^§^ |  | 148 | -0.118 (0.065) | 0.069 |  |  |  | *N.A.* |  |  |  | *N.A.* |
| Ever taken hormone replacement therapy^‡^ |  | 147 | -0.014 (0.050) | 0.773 |  | 54 |  | *N.D.* |  | 93 | -0.013 (0.029) | 0.654 |
| Benign breast disorder^‡^ |  | 150 | 0.018 (0.046) | 0.692 |  | 53 | -0.001 (0.134) | 0.996 |  | 94 | 0.038 (0.030) | 0.204 |

Abbreviations: BMI, body mass index; N.A., not applicable; N.D., not determined; SE, standard error; y, years.

* Log transformed variable

^a^ Unadjusted model

^b^ Adjusted for age

^¶^ Parous versus nulliparous

^◊^ Minimum total duration of breast-feeding (months) among parous women

^‡^ Ever versus never

^§^ Postmenopausal versus premenopausal

**Supplemental Table S6.** Linear regression analysis of adipose area by risk factors, for all women and stratified by menopausal status, adjusted for age and BMI.

|  |  | Adipose area (%)  All women (*N*=153) | | |  | Adipose area (%)  Premenopausal women (*N*=55) | | |  | Adipose area (%)  Postmenopausal women (*N*=95) | | |
| --- | --- | --- | --- | --- | --- | --- | --- | --- | --- | --- | --- | --- |
| Variables |  | *N* | Estimates β (SE) | *P* |  | *N* | Estimates β (SE) | *P* |  | *N* | Estimates β (SE) | *P* |
| Age at biopsy (y) |  | 153 | -0.090 (0.280) | 0.747^a^ |  | 55 | -0.897 (0.882) | 0.314^a^ |  | 95 | 0.012 (0.515) | 0.982^a^ |
| BMI (Kg/m^2^) |  | 151 | 1.986 (0.566) | **0.001^b^** |  | 54 | 2.573 (0.854) | **0.004**^b^ |  | 94 | 1.661 (0.767) | **0.033**^b^ |
| Age at menarche (y) |  | 144 | 0.022 (1.809) | 0.990 |  | 53 | -3.745 (2.625) | 0.160 |  | 91 | 2.809 (2.464) | 0.257 |
| Age at first birth (y) |  | 126 | -0.477 (0.503) | 0.345 |  | 47 | 0.068 (0.668) | 0.919 |  | 79 | -1.080 (0.757) | 0.158 |
| Age at menopause (y) |  |  |  | *N.A.* |  |  |  | *N.A.* |  | 78 | 0.445 (0.750) | 0.555 |
| Parous status^¶^ |  | 145 | 4.072 (7.394) | 0.583 |  | 54 | 11.554 (11.519) | 0.321 |  | 91 | 0.650 (9.770) | 0.947 |
| Pregnancies (number) |  | 145 | -1.406 (1.586) | 0.377 |  | 54 | -2.863 (2.539) | 0.236 |  | 91 | -0.485 (2.064) | 0.815 |
| Births (number) |  | 145 | -0.706 (2.362) | 0.765 |  | 54 | 0.436 (3.661) | 0.906 |  | 91 | -1.209 (3.132) | 0.700 |
| Breast-feeding (months)^◊^ |  | 120 | -0.554 (0.279) | **0.050** |  | 44 | -0.489 (0.462) | 0.296 |  | 76 | -0.615 (0.360) | 0.091 |
| Ever taken oral contraceptives^‡^ |  | 143 | -4.142 (6.980) | 0.554 |  | 54 | -17.151 (16.220) | 0.295 |  | 89 | 0.570 (8.219) | 0.945 |
| Postmenopausal status^§^ |  | 148 | 3.123 (7.919) | 0.694 |  |  |  | *N.A.* |  |  |  | *N.A.* |
| Ever taken hormone replacement therapy^‡^ |  | 147 | 4.493 (6.009) | 0.456 |  | 54 |  | *N.D.* |  | 93 | 5.991 (6.858) | 0.385 |
| Benign breast disorder^‡^ |  | 150 | 0.786 (5.604) | 0.889 |  | 53 | -3.540 (10.233) | 0.731 |  | 94 | 2.354 (7.060) | 0.740 |

Abbreviations: BMI, body mass index; N.A., not applicable; N.D., not determined; SE, standard error; y, years.

^a^ Unadjusted model

^b^ Adjusted for age

^¶^ Parous versus nulliparous

^◊^ Minimum total duration of breast-feeding (months) among parous women

^‡^ Ever versus never

^§^ Postmenopausal versus premenopausal
